# Effect of low glycemic index food and postprandial exercise on blood glucose level, oxidative stress and antioxidant capacity

**DOI:** 10.3892/etm.2015.2228

**Published:** 2015-01-28

**Authors:** NORIAKI KASUYA, SHOICHIRO OHTA, YOSHIKAZU TAKANAMI, YUKARI KAWAI, YUTAKA INOUE, ISAMU MURATA, IKUO KANAMOTO

**Affiliations:** 1 Laboratory of Drug Safety Management, Division of Pharmaceutical Sciences, Josai University, Sakado, Saitama 350-0295, Japan; 2 Department of Urology, Kan-Etsu Hospital, Tsurugashima, Saitama 350-2213, Japan; 3 Department of Food Science, Otsuma Women’s University, Tokyo 102-8357, Japan; 4 Louis Pasteur Center for Medical Research, Kyoto 606-8225, Japan

**Keywords:** glycemic index, blood glucose level, oxidative stress, antioxidant capacity

## Abstract

Low glycemic index (GI) food and postprandial exercise are non-drug therapies for improving postprandial hyperglycemia. The present randomized, crossover study investigated the effect of low GI food combined with postprandial exercise on postprandial blood glucose level, oxidative stress and antioxidant capacity. A total of 13 healthy subjects were each used in four experiments: i) rice only (control), ii) salad prior to rice (LGI), iii) exercise following rice (EX) and iv) salad prior to rice and exercise following rice (MIX). The blood glucose level, oxidative stress and antioxidant capacity were then measured. At 60 min after the meal, the blood glucose level was observed to be increased in the MIX group compared with that in the LGI group. Furthermore, at 180 min, the antioxidant capacity was found to be reduced in the MIX group compared with those of the LGI and EX groups. These findings suggest that low GI food combined with postprandial exercise does not improve postprandial hyperglycemia. It may be necessary to establish optimal timing and intensity when combining low GI food with postprandial exercise to improve postprandial hyperglycemia.

## Introduction

The number of individuals with diabetes has been increasing rapidly worldwide. Poorly controlled diabetes leads to numerous complications (1,2), including macroangiopathy, which is a major cause of mortality in patients with diabetes (3). One independent risk factor for macroangiopathy in patients with diabetes is postprandial hyperglycemia, which has been proposed to be caused by the induction of oxidative stress (5–9). In order to prevent the onset and/or progression of macroangiopathy in patients with diabetes, it is important to suppress hyperglycemia after meals. Two non-drug treatments for this purpose include the intake of food with a low glycemic index (GI) and postprandial exercise. These treatments have been investigated separately in several studies; however, to the best of our knowledge, they have not been investigated as a combined treatment. In the present study, the effectiveness of the combination of low-GI food intake and postprandial exercise for the suppression of hyperglycemia was investigated through the monitoring of blood glucose levels, oxidative stress and antioxidative activity.

## Materials and methods

### Subjects and treatment

Nine male and four female individuals (range, 20–29 years old) who had no abnormal glucose tolerance in medical check-ups for the prior year and who had received no drug therapy were enrolled in the present study (Table I). Female subjects did not undergo the present study during their menstrual periods.

For a test meal, cooked rice was prepared. The quantity of saccharide in each test meal was set at 50 g through adjusting the quantity of cooked rice provided in the meal. As a low-GI meal, a vegetable salad of shredded cabbage dressed with olive oil, vinegar and salt was prepared (Table II).

For the postprandial exercise, step aerobics at a speed of 80 steps per min was performed. The following four experiments were randomly assigned to the 13 subjects, which were conducted using the crossover method: i) consumption of the cooked rice only (control; n=13); ii) consumption of the vegetable salad first and then the cooked rice (LGI; n=13); iii) consumption of the cooked rice and performing the exercise 30 min later (EX; n=13); and iv) consumption of the vegetable salad first, followed by the cooked rice and performing the exercise 30 min later (MIX; n=13). The day prior to the test, food and drink other than water was prohibited after 9:00 p.m. The starting time of consuming the cooked rice or the vegetable salad (whichever was first) was defined as 0 min. When the vegetable salad was consumed first, the cooked rice was consumed 10 min later. The test meals were consumed at the individual’s own pace. All participants provided written informed consent, and the study protocol was approved by the Ethics Committee of Josai University (Sakado, Japan).

### Blood sample collection and analysis of glucose and insulin levels

Blood samples were taken from the cutaneous vein of the fingertip using a puncture device for self exsanguination eight times: 10 min prior to the meal, then 20, 30, 45, 60, 90, 120 and 180 min following the first meal. Blood was collected using a capillary tube and blood plasma was obtained through centrifugation.

Blood glucose levels were measured using a self-administered blood glucose measuring device (Glutest Neo Super^®^; Sanwa Kagaku Kenkyusho Co. Ltd., Aichi, Japan). Plasma insulin values in 25 μl plasma were measured using an insulin measuring kit (YK060 Insulin ELISA kit^®^; Yanaihara Institute Inc., Shizuoka, Japan). Levels of derivatives of reactive oxidative metabolites (d-ROM; an oxidative stress marker) and the biological antioxidant potential (BAP; a marker of antioxidative activity ) were measured in 30 μl plasma using a free radical analyzer (FREE Carpe Diem; Wismerll, Tokyo, Japan) at 0, 60, 120 and 180 min. Four of the 13 subjects provided insufficient sample for measuring d-ROM and BAP.

### Statistical analysis

Sequential blood glucose, insulin, d-ROM and BAP levels recorded for the subjects following the consumption of the test meal were set as the Δblood sugar, Δinsulin level, Δd-ROM level and ΔBAP level by subtraction of the level at 0 min. ΔBAP was divided by Δd-ROM, then divided by 7.541. This result was designated as the Δmodified BAP/d-ROM ratio, which was considered to be an indicator of antioxidant capacity. The sequential changes in blood glucose and plasma insulin levels were assessed in each group. P<0.05 was considered to indicate a statistically significant difference between groups. The data were analyzed using Statcel2 software (OMS Publishing Inc., Saitama, Japan). The results were assessed with Tukey Kramer test.

## Results

### Changes in blood glucose and insulin levels

**Figure 1:**
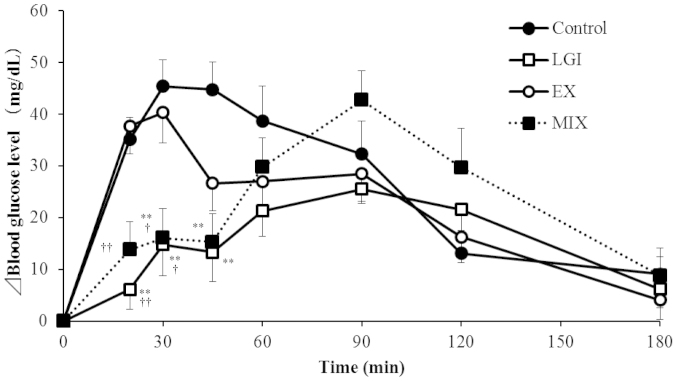
Mean changes in Δblood glucose. Data are presented as the mean ± standard error of the mean (n=13). ^**^P<0.01 vs. control; ^†^P<0.05 vs. EX; ^††^P<0.01 vs. EX.

**Figure 2:**
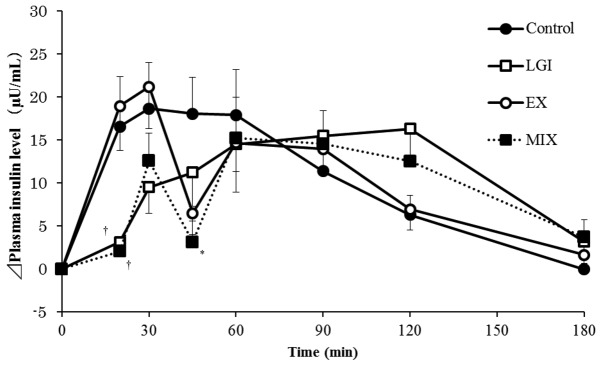
Mean changes in Δplasma insulin. Data are presented as the mean ± standard error of the mean (n=13). ^*^P<0.05 vs. control; ^†^P<0.05 vs. EX.

The changes in blood glucose levels in each group are shown in Fig. 1. The blood glucose levels in the individuals in the control group were found to increase immediately following the meal and reached a peak at 30 min following the meal, then decreased. In the LGI group, the blood glucose level was observed to increase for up to 45 min following the meal, but it was significantly suppressed compared with that in the control group. In the EX group, the increase in blood glucose levels between 45 and 60 min following the meal was suppressed compared with that in the control group. In the MIX group, the changes in blood glucose levels up to 45 min following the meal were comparable with those in the LGI group; however, the blood sugar level at the 60 min time-point was increased compared with that in the LGI group. The sequential changes in insulin values following the meal in each group were similar to those in the blood glucose levels (Fig. 2). However, the insulin values in the EX and MIX groups were found to be decreased 45 min following the meal compared with those in the control group.

### Time course of antioxidant potential

**Figure 3:**
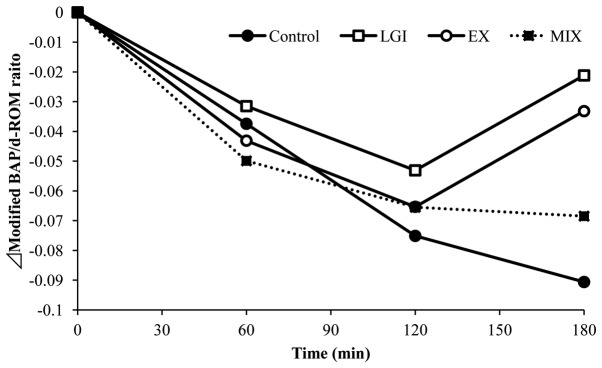
Mean changes in Δmodified BAP/d-ROM ratio. Data are presented as the mean ± standard error of the mean (n=9).

The antioxidant potential of each of the test groups is shown in Fig. 3. The Δmodified BAP/d-ROM ratio in the control group was observed to decrease over time. By contrast, the reduction in this ratio was suppressed in the LGI group compared with that in the control group. Moreover, at 180 min, the Δmodified BAP/d-ROM ratio in the control group was found to exhibit the greatest reduction amongst all the groups, while the Δmodified BAP/d-ROM ratio in the LGI and EX groups was increased compared with that at 120 min. The Δmodified BAP/d-ROM ratio in MIX group was not observed to increase at 180 min.

## Discussion

The results indicate that the combination of low GI food consumption and post-meal exercise affects postprandial blood glucose. Blood glucose data revealed that combining low-GI food intake and 10 min of exercise 30 min following a meal increases postprandial blood glucose levels compared with those observed following only low-GI food consumption. Regarding the effect of low-GI food on postprandial blood glucose, the low-GI meal was found to have a suppressive effect on the rapid increase in postprandial blood glucose levels. A meta-analysis of studies of this effect revealed that this benefit of low-GI food reduces several cardiovascular risk factors (10).

In the present study, a vegetable salad was used as the low-GI food. The data from the treatment and control groups revealed that consuming the salad prior to the cooked rice effectively helped control postprandial blood glucose levels, as observed in a previous study (11). It has been reported that uncooked cabbage, olive oil and vinegar (the components of the salad used in the present study) each suppress hyperglycemia following a meal. The water-soluble dietary fiber in cabbage has high viscosity, which slows down the rate of gastric emptying and this delay has been proposed to suppress the rapid elevation in glycemia (12). The insoluble dietary fiber in cabbage has also been reported to improve the insulin resistance of the body (13). Olive oil contains a high quantity of monounsaturated fatty acids that enhance insulin sensitivity and lower postprandial blood glucose through stimulating the secretion of glucagon-like peptide-1 (14). Vinegar has also been proposed to lower the gastric emptying rate and thus slow down digestion, suppressing hyperglycemia following a meal (15). These characteristics of low-GI foods are supported by the findings of the present study.

Regarding the effect of post-meal exercise on postprandial blood glucose, Larsen *et al* (16) observed that performing moderate exercise 45 min after breakfast lowered blood glucose and/or insulin levels in patients with diabetes. The underlying mechanism may involve an increase in glucose consumption in the skeletal muscle (16). Viollet *et al* (17) reported that adenosine monophosphate-activated protein (AMP) kinase is activated due to the increase in the AMP/ATP ratio upon exercise in the skeletal muscle, and glucose transporter type 4 becomes transferred to the cell membrane. However, it has also been reported that if an individual exercises in a fasting state, the glucose supply from the liver is stimulated as catecholamine secretion is increased, which is associated with sympathetic nerve stimulation; furthermore, insulin secretion, which suppresses gluconeogenesis in the liver, is decreased (18).

In the present study, the EX group exhibited an inhibited postprandial blood glucose increase at 45 and 60 min following the meal compared with the glucose level in the control group. The MIX group exhibited a greater increase in postprandial blood glucose level compared with that of the LGI group. In light of the aforementioned mechanism, the reason why the MIX group showed a greater increase in postprandial blood sugar level than the LGI group may be that the exercise was performed when the blood sugar level was not sufficiently high; thus, glucose was supplied from the liver.

An increase in blood glucose levels has been proposed to lead to oxidative stress. Ceriello *et al* (19) observed that patients with diabetes exhibit a significant post-meal reduction in antioxidant levels and an increase in the levels of malondialdehyde, a marker of oxidative stress. In the present study, the antioxidant capacity of the control group continued to decrease until 180 min after the meal, whereas the antioxidant capacity in the LGI and EX groups was beginning to increase at 180 min following the meal. A previous study indicated that the oxidation of LDL is prevented by the polyphenols contained in olive oil (20), and the exercise-induced reduction in the manifestation of NADPH oxidase (21) is thought to contribute to this.

It is well established that a controlled diet and exercise are essential therapies for the treatment of diabetes. Thus, the consumption of low-GI food combined with post-meal exercise may be used as a treatment option for patients with diabetes. The findings of the present study support the use of dietary counseling regarding low-GI food. However, the individuals employed in the present study were healthy, with no diabetes; thus, the data obtained should be investigated in further studies of patients with diabetes.

## Figures and Tables

**Table I:** Subject characteristics.

Parameter	Total (n=13)	Male (n=9)	Female (n=4)
Age (years)	22.8±1.7	23.0±2.0	22.3±1.0
Height (cm)	168.2±9.0	172.3±6.8	158.8±5.5
Weight (kg)	60.6±8.3	63.9±7.6	53.3±4.1
BMI (kg/m^2^)	21.4±1.5	21.5±1.7	21.1±0.8

Data are presented as the mean ± standard deviation. BMI, body mass index.

**Table II:** Nutrient composition and quantity of the test foods.

Food	Weight (g)	Protein (g)	Fat (g)	Carbohydrate (g)	Energy (kcal)
White rice	147.0	3.1	0.6	50.0	223.2
Cabbage	60.0	0.8	0.1	3.1	13.8
Olive oil	10.0	0.0	10.0	0.0	92.1
Vinegar	10.0	0.0	0.0	0.2	25.0
Total	227.0	3.9	10.7	53.3	354.1
